# Development of an LFD-RPA Assay for Rapid Detection of *Pentatrichomonas hominis* Infection in Dogs

**DOI:** 10.3390/cimb45110579

**Published:** 2023-11-17

**Authors:** Yao Rong, Xichen Zhang, Xuejiao Chen, Jianhua Li, Pengtao Gong, Xiaocen Wang, Xin Li, Xu Zhang, Taotao Yue, Hongbo Zhang, Xiaofei Zhou, Nan Zhang

**Affiliations:** Key Laboratory of Zoonosis Research of Ministry of Education, Institute of Zoonosis, College of Veterinary Medicine, Jilin University, Changchun 130062, China; rongyao21@mails.jlu.edu.cn (Y.R.); xczhang@jlu.edu.cn (X.Z.); chenxj981001@163.com (X.C.); jianhuali7207@163.com (J.L.); gongpt@jlu.edu.cn (P.G.); wangxiaocen2016@163.com (X.W.); jlulixin0928@163.com (X.L.); zhangxu1311@163.com (X.Z.); taotaoyue1996@163.com (T.Y.); zhanghb320563@163.com (H.Z.); xfzhou@jlu.edu.cn (X.Z.)

**Keywords:** *Pentatrichomonas hominis*, zoonotic pathogen, LFD-RPA, dogs, rapid detection

## Abstract

*Pentatrichomonas hominis* is a trichomonad protozoan that infects the cecum and colon of humans and other mammals. It is a zoonotic pathogen that causes diarrhea in both animals and humans. As companion animals, dogs infected with *P. hominis* pose a risk of transmitting it to humans. Current methods, such as direct smears and polymerase chain reaction (PCR), used for *P. hominis* detection have limitations, including low detection rates and the need for specialized equipment. Therefore, there is an urgent need to develop rapid, sensitive, and simple detection methods for clinical application. Recombinase polymerase amplification (RPA) has emerged as a technology for rapid pathogen detection. In this study, we developed a lateral flow dipstick (LFD)-RPA method based on the highly conserved *SPO11-1* gene for detecting *P. hominis* infection by optimizing the primers, probes, and reaction conditions, and evaluating cross-reactivity with genomes of *Giardia duodenalis* and other parasites. The LFD-RPA method was then used to test 128 dog fecal samples collected from Changchun. The results confirmed the high specificity of the method with no cross-reactivity with the five other parasites. The lowest detection limit of the method was 10^2^ copies/µL, and its sensitivity was 10^0^ times higher than that of the conventional PCR method. Consistent with the positivity rate observed using nested PCR, 12 samples (out of 128) tested positive using this method (positivity rate, 9.38%). In conclusion, the LFD-RPA method developed in this study represents a simple and sensitive assay that allows for the rapid detection of *P. hominis* infection in dogs, especially in this field.

## 1. Introduction

*Pentatrichomonas hominis* (*P. hominis*) is a zoonotic parasite that primarily colonizes the colon and cecum in humans and other vertebrates and is distributed worldwide. It was previously considered to be an intestinal non-pathogenic symbiotic parasite or a conditional pathogenic protozoan. However, in recent years, *P. hominis* has been isolated from human and animal feces with diarrhea [1], indicating that it may be pathogenic. *P. hominis* infection causes mushy feces, diarrhea, constipation, bloody stools, and even rectal prolapse in animals [2]. Recent studies have shown that *P. hominis* infections are associated with gastrointestinal cancer [3].

*P. hominis* has a relatively wide host range, and has been detected in various animals, including dogs and cats, and in humans [4,5,6,7]. *P. hominis* that infects both dogs and humans belongs to the CC1 type [8]. This suggests that *P. hominis* can spread between humans and dogs. Dogs, as companion animals, are often in close contact with humans, and hence those infected with *P. hominis* may transmit it to humans. Current methods for detecting *P. hominis* infection in animals mainly include pathogen detection and polymerase chain reaction (PCR). Although the direct smear method is the “gold standard” for detecting infection, it has a low detection rate and pathogen detection is easy to miss [9]. PCR is more specific and sensitive than pathogen detection; however, it requires special equipment, and is not practical for field application [10]. Although rapid detection methods for some parasite infections including colloidal gold and RPA, etc., have been established, there are no rapid detection methods for *P. hominis* infection currently. Therefore, it is necessary to establish a rapid, sensitive, convenient, and suitable method for the clinical detection of *P. hominis* infection.

Recombinase polymerase amplification (RPA) is a relatively new and rapid diagnostic method that has been widely used for the detection of parasites [11,12]. Lateral flow dipstick (LFD)-RPA is a rapid, on-site detection system that combines RPA technology with LFD. Currently, there are no reports on an LFD-RPA-based detection method for *P. hominis* infection. Therefore, in this study, we developed an LFD-RPA method using the target gene *SPO11-1* for the clinical detection of *P. hominis* infection in dogs.

## 2. Materials and Methods

### 2.1. Sample Collection

A *P. hominis* sample was collected from the feces of naturally-infected dogs from the endemic area of Changchun (Jilin Province, China). Several *P. hominis*-related and other common parasites, including *Giardia duodenalis* (*G. duodenalis*), *Tritrichomonas foetus* (*T. foetus*), *Isospora canis* (*I. canis*), *Toxocara canis* (*T. canis*), and *Cryptosporidium parvum* (*C. parvum*) (all maintained in our laboratory) were used as controls to evaluate the specificity of this method. Dog fecal samples used for analyses were randomly collected from a stray animal rescue station and an animal hospital in Changchun. The collection procedures of dog feces were conducted in strict accordance with the guidelines of the Animal Care and Welfare Committee of Jilin University (No: SY202201109).

### 2.2. DNA Extraction

Total genomic DNA was extracted from fecal samples (from dogs with *P. hominis* infection) using the TIANamp Genomic DNA Kit (Tiangen, Beijing, China) following the manufacturer’s instructions. Genomic DNA was extracted from the other control samples using the same method and stored at −20 °C.

### 2.3. Primer and Probe Design

*SPO11-1* (GenBank accession no. JF298016) was selected as the target gene for the detection of *P. hominis* infection using LFD-RPA. Twelve pairs of primers were designed using Primer 5.0 software (Table 1). PCR amplification followed by electrophoresis on a 3% agarose gel was used to screen for high-specificity primers. The genomes of *G. duodenalis*, *Trichomonas murinus*, *Trichomonas vaginalis*, and *P. hominis* were used to validate the primer specificity. Based on the target fragment amplified by the primers, the DNA probe was designed with the addition of 6-FAM modification at the 5′ end and C3-spacer modification at the 3′ end (Table 2). Using *P. hominis* as the positive template, the two probes were compared in the LFD-RPA assay via incubation in a 39 °C water bath for 30 min and observation of the color change of the LFD test line.

### 2.4. RPA Reaction and Lateral Flow Reading

RPA reactions were performed using the RAA-nfo Nucleic Acid Amplification Kit (Hangzhou ZC Bio-Sci & Tech Co., Ltd., Hangzhou, China) according to the manufacturer’s instructions. A reaction mixture consisting of 25 µL of A Buffer, 1.2 µL of 2 µM upstream primer, 1.2 µL of 2 µM downstream primer, 0.25 µL of 2 µM probe, and 15.1 µL of ddH_2_O was added to the reaction dry powder tube. After mixing, the mixture was divided into two tubes, and 2.5 µL of DNA sample and 1.25 µL of B Buffer were added to each tube. After centrifugation at low speed, the mixture was mixed and allowed to settle at the bottom.

The tubes were incubated in a water bath at six different temperatures (25, 30, 35, 40, 45, and 50 °C) for 20 min. The amplification product was diluted 8× to 225 µL, and 10 µL of the amplification product was placed in the sample pad of the disposable Nucleic Acid Detection Kit (Hangzhou ZC Bio-Sci & Tech Co., Ltd.). Then, 10 µL of LFD buffer was added, and the color change of the LFD was observed after 2–3 min. The optimal reaction temperature was determined based on the color intensity of the LFD test line.

To determine the optimal reaction time, the water bath was heated to the optimal reaction temperature, and the LFD-RPA reactions were performed for different periods (0, 2, 5, 10, 15, 20, 25, or 30 min). The color intensity of the LFD test line was observed.

### 2.5. Specificity of the LFD-RPA Assay

To verify the specificity of the LFD-RPA assay, the genomes of *G. duodenalis*, *T. foetus*, *I. canis*, *T. canis*, and *C. parvum* were used as RPA reaction templates, and LFD-RPA was performed at the optimal reaction time and temperature. The color intensity of the LFD test line was observed to determine the specificity of the detection method.

### 2.6. Sensitivity of the LFD-RPA Assay

Genomic DNA of *P. hominis* was serially diluted 10-fold (108–100 copies/μL), and different copy numbers of the genome were used as templates for LFD-RPA. The minimum detection limit of the method was determined via observation of the color intensity of the LFD test line, and nucleic acid templates were detected with traditional PCR using the designed RPA primers (Table 3). The PCR program was as follows: 95 °C for 3 min; thirty-four 3-step cycles consisting of denaturation at 95 °C for 30 s, annealing at 61.1 °C for 30 s, and extension at 72 °C for 20 s; followed by final extension at 72 °C for 10 min. The minimum detectable amount with this primer for *P. hominis* in the PCR assay was determined to compare the sensitivity of the LFD-RPA method.

### 2.7. Applicability of the LFD-RPA Assay

A total of 128 clinical fecal samples from dogs were obtained from an animal rescue station and an animal hospital in Changchun. *P. hominis* infection status was determined using the LFD-RPA method, based on the color intensity of the LFD test line. A nested PCR [13] and microscopic examination were performed simultaneously on these samples, and the results were analyzed and compared with those of the LFD-RPA method. The primers and program used for nested PCR are listed in Table 4 and Table 5. The reaction conditions were as follows: first round of nested PCR: 95 °C for 3 min, followed by 34 cycles at 95 °C for 30 s, 55 °C for 30 s, and 72 °C for 38 s, followed by final 72 °C extension for 7 min. Second round of nested PCR: 95 °C for 3 min, followed by 40 cycles at 95 °C for 45 s, 57 °C for 30 s, and 72 °C for 1 min, followed by final 72 °C extension for 7 min.

## 3. Results

### 3.1. Primer and Probe Design

**Figure 1:**
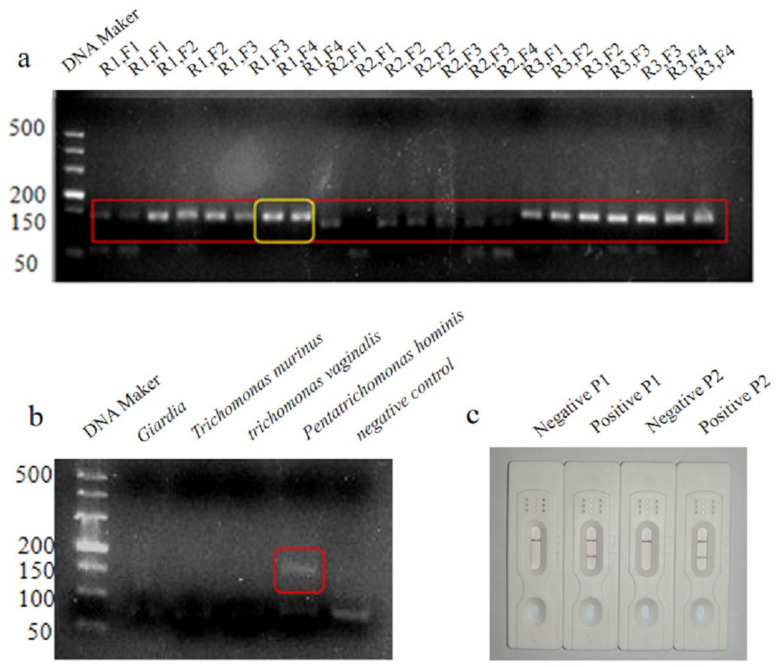
Results of agarose gel electrophoresis with 12 pairs of designed primers, The red square is the target fragment and the yellow square is the best target fragment that we choose to use as the best primer pair. (**a**); primer specificity verification results, Shown in the red square is an amplified fragment of *pentacomonas hominis*. (**b**); results of the LFD assay for comparison of the two probes (**c**).

Twelve pairs of primers were designed for PCR, and the amplification products were screened using 3% agarose gel electrophoresis. Primers R1 and F4 were selected as the upstream and downstream primers, respectively, for LFD-RPA, as they produced bright bands, exhibited strong specificity, and did not generate primer dimers (Figure 1a). The results of the specificity verification assay showed that the primers amplified only the genome of P. hominis and not that of the other parasite genomes tested, thus confirming that the primers were highly specific (Figure 1b). Based on the target fragment amplified by the primers, two probes were designed and the intensity of the test line produced by the two probes was compared. Based on the results, probe P1 was selected as the final probe (Figure 1c).

### 3.2. Optimization of the LFD-RPA Assay

**Figure 2:**
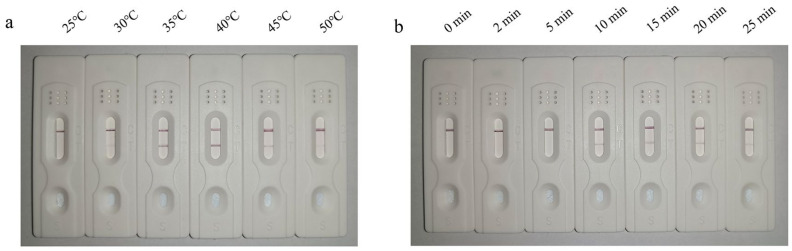
LFD-RPA showing detectable test line at 35 °C that gradually faded at 45 °C (**a**). LFD-RPA reaction performed at the optimal reaction temperature for 10 min showing initiation of amplification as evidenced by clearly detectable test line in the LFD (**b**).

The reaction temperature was optimized by performing the reaction for 20 min at different temperatures. The results showed the appearance of a faint test line at 30 °C, which increased in intensity at 35 °C. The color intensity started to reduce at 45 °C and became even fainter at 50 °C. Therefore, 35 °C was determined to be the optimal reaction temperature (Figure 2a).

The reaction time was optimized by performing the reaction at the optimal reaction temperature of 35 °C for different time periods. The results showed an absence of amplification at 0–5 min, as the test line in the LFD was undetectable. The amplification reaction began at a reaction time of 10 min, as evidenced by the appearance of a detection line in the dipsticks. Therefore, the optimal reaction time was determined to be 10 min (Figure 2b).

### 3.3. Optimization of the LFD-RPA Assay

**Figure 3:**
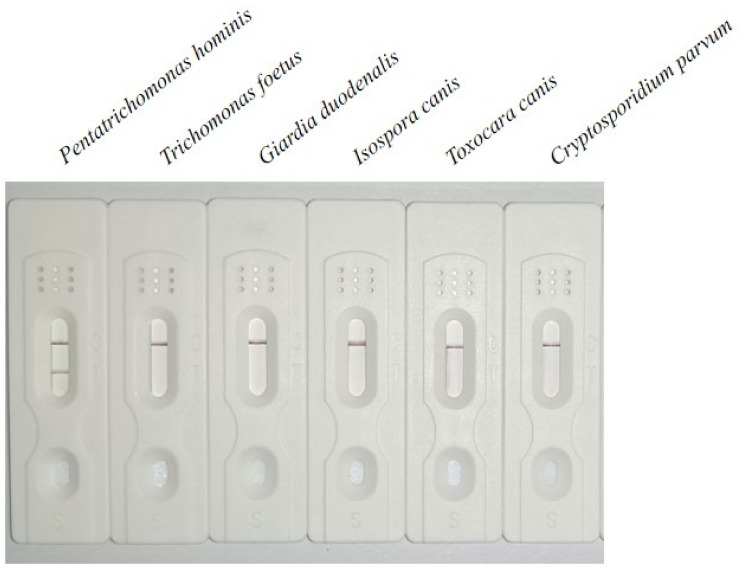
Specificity analysis of LFD-RPA showing specific amplification of the *P. hominis* genome.

The genomes of *P. hominis*, *G. duodenalis*, *T. foetus*, *I. foetus*, *T. canis*, and *C. parvum* were used as templates to test the specificity of the LFD-RPA. The reaction was performed at the optimal reaction time and temperature. The results showed that only the genome was amplified using the test strip. No amplification was observed for any of the other parasites, indicating optimal specificity of the assay (Figure 3).

### 3.4. Sensitivity Analysis of LFD-RPA Assay

**Figure 4:**
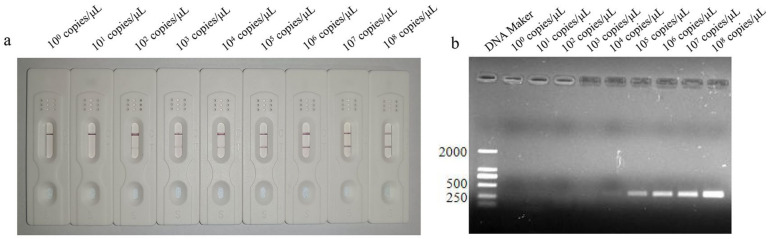
Lowest detection limit of LFD-RPA method (appearance of test line) at 10^2^ copies/μL (**a**), and the lowest detection limit of PCR method (appearance of corresponding band) at 10^4^ copies/μL (**b**).

The sensitivity of the LFD-RPA was analyzed using the different copies of *P. hominis* genomic samples. The results showed that the lowest detection limit of the LFD-RPA method was 10^2^ copies/μL (Figure 4a), whereas as that of PCR was 10^4^ copies/μL (Figure 4b), showing that the sensitivity of the LFD-RPA is higher than that of PCR.

### 3.5. Comparative Clinical Samples

**Figure 5:**
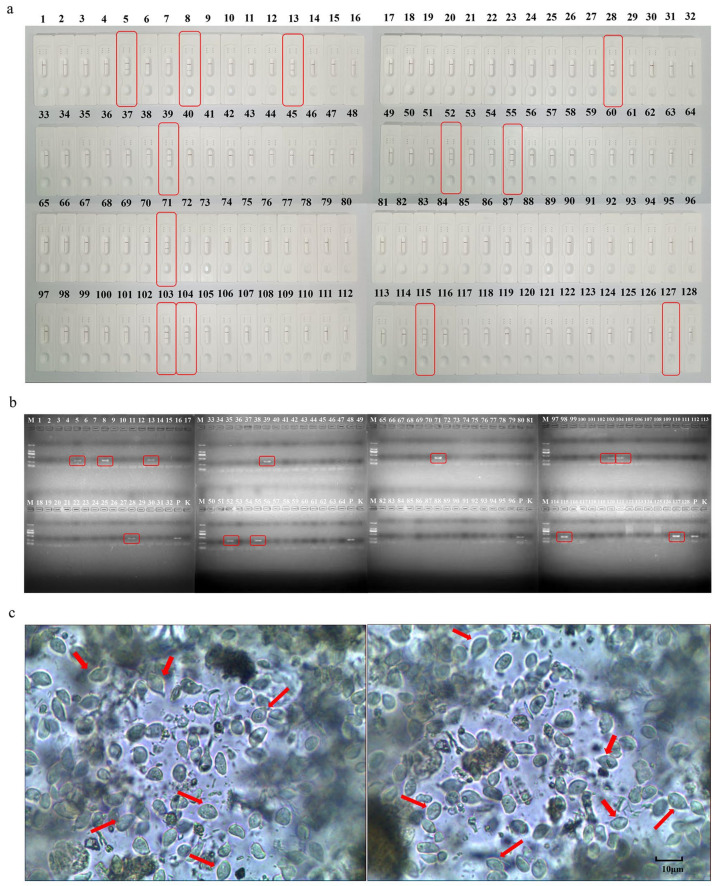
Detection of 12 positive samples (out of 128 dog fecal samples tested) using LFD-RPA, the red squares show positive samples. (**a**); detection of 12 positive samples (out of 128 dog fecal samples tested) using nested PCR assay. P, positive control; K, negative control, the red squares show positive samples. (**b**). Partial results of stool microscopy, the red arrow points to the body of *pentrichomonas hominis* as seen under the microscope. (**c**).

The LFD-RPA method was used to analyze 128 clinical dog fecal samples. The results showed that with the LFD-RPA method, 12 out of 128 fecal samples tested positive with a positivity rate of 9.38% (Figure 5a); that was consistent with the results of nested PCR (Figure 5b). Thus, the results of the LFD-RPA were consistent with those of nested PCR (Figure 5c), with a coincidence rate of 100%.

## 4. Discussion

Recent molecular epidemiological studies indicate a high rate of *P. hominis* infection among dogs in many countries, with infection rates ranging from 6.45 to 47.4% [4,5,6]. This poses a high risk of zoonotic transmission to humans. In addition, *P. hominis* has been detected in the feces of immuno-compromised patients, including those with rheumatoid arthritis treated with adalimumab, irritable bowel syndrome, and empyema [14,15,16]. However, methods for the detection of *P. hominis* infection are limited to traditional pathogen- and PCR-based detection modalities.

LFD-RPA has been widely used for the detection of bacteria such as *Brucella*, viruses such as H7N9, IBRV [17,18,19], and parasites. Crannell et al. [20] developed an RPA assay for the detection of *G. duodenalis* infection based on the amplification of a fragment of the *β-giardin* gene. Li et al. [21] developed an LFD-RPA method using the highly conserved mitochondrial small-subunit ribosomal RNA as the target gene to detect *Trichinella spiralis* infection. SPO11 is a member of a highly conserved protein family, and its sequence, structure and functions are highly conserved [22]. SPO11 has been extensively studied in plants. Studies of *Arabidopsis thaliana* and *wheat* have shown that SPO11 controls the formation and repair of meiosis-specific DNA double-strand breaks (DSBs), and its function has been conserved during biological evolution [23,24]. It catalyzes specific DNA DSBs during meiosis in *Saccharomyces cerevisiae*, *fission yeast*, and *Caenorhabditis elegans*, and plays an important role in the sexual reproduction of organisms [25]. In this study, the *SPO11-1* gene was selected as the specific target for the detection of *P. hominis* infection.

Screening of suitable primers and probes is crucial for the success of RPA. Unlike in basal-RPA systems, endonuclease Ⅳ (Nfo), nfo probes, and primers labeled with biotin or digoxin were used in the LFD-RPA reaction [26]. The probe contained a base sequence with a marker at the 5′ end, a tetrahydrofuran (THF), and a blocking modification group at the 3′ end. Nfo, a DNA repair enzyme, recognizes and cleaves this site. As the reaction proceeds, the cleaved probe and downstream primer form a double-labeled amplicon with both the probe and prime-specific labels. The RPA primer sequence selected for the method was from a highly differentiated region of *SPO11-1*, and 6-FAM (at the 5′ end) and C3-spacer (at the 3′ end) were selected as optimal modifications.

The sensitivity of RPA is markedly higher than that of PCR. A previous report showed that the minimum detection limit is 0.5 *Cryptosporidium oocysts* for LFA-RPA to detect *Cryptosporidium* infection in dairy cows, and its sensitivity is 6× that of nested PCR [27]. Another study used LFD-RPA to detect the three stages of *Clonorchis sinensis*, including adults, eggs, and cercariae, and reported a minimum detection limit of 10 fg for adults with a sensitivity 1000× that of PCR. The detection limit for purified eggs and metacercariae was one, and the sensitivity was 25× that of PCR [28]. We used SPO11-1 as the target gene to develop an LFD-RPA assay to detect *P. hominis* infection. The minimum detection limit of the assay was 102 copies/μL, whereas that of PCR using the same primers was 104 copies/μL. The sensitivity of the LFD-RPA assay was 100× that of PCR.

## 5. Conclusions

In this study, we successfully developed an LFD-RPA assay based on the *SPO11-1* gene for the detection of *P. hominis* infection. Screening of clinical samples showed that the LFD-RPA method is fast, simple, and has an easy readout, which renders it useful for clinical application in the rapid detection of *P. hominis* infection in dogs.

## Figures and Tables

**Table 1:** Primer design for *SPO11-1* gene.

Name	Sequence (5′ to 3′)
Ph-F1-SPO11	CAGATAGTGGTGGACGAGCCCTGGATTGCC
Ph-F2-SPO11	AGATAGTGGTGGACGAGCCCTGGATTGCCT
Ph-F3-SPO11	GATAGTGGTGGACGAGCCCTGGATTGCCTT
Ph-F4-SPO11	ATAGTGGTGGACGAGCCCTGGATTGCCTTG
Ph-R1-SPO11	CTTGTGCCAATCTCATAAAAACGCTCTCCT
Ph-R2-SPO11	CGCTCTCCTTTTCAACAACAATAACCGCAA
Ph-R3-SPO11	GATGCTTGTGCCAATCTCATAAAAACGCTC

**Table 2:** Primer probe sequence.

Name	Sequence (5′ to 3′)
SPO11-F4-RPA	ATAGTGGTGGACGAGCCCTGGATTGCCTTG
SPO11-R1-RPA	Biotin-CTTGTGCCAATCTCATAAAAACGCTCTCCT
P1	6-FAM-gacgtgtagttccaataccagcttcaccagatg-THF-cataaaagcagttagatgtaca-C3 Spacer
P2	6-FAM-ccaataccagcttcaccagatggcataaaagcagtta-THF-atgtacaggaattgcggttatt-C3 Spacer

**Table 3:** PCR system.

Reagent	Dosage
ddH_2_O	37.75 μL
10× PCR Buffer (Mg^2+^ plus)	5 μL
dNTP Mixture	4 μL
Forward Primer	1 μL
Reverse Primer	1 μL
rTaq	0.25 μL
DNA	1 μL

**Table 4:** Primer sequences.

Name	Sequence (5′ to 3′)
18SrRNA-F1	ATGGCGAGTGGTGGAATA
18SrRNA-R1	CCCAACTACGCTAAGGATT
18SrRNA-F2	TGTAAACGATGCCGACAGAG
18SrRNA-R2	CAACACTGAAGCCAATGCGAGC

**Table 5:** Nested PCR system.

Reagent	Dosage
ddH_2_O	37.75 μL
10× PCR Buffer (Mg^2+^ plus)	5 μL
dNTP Mixture	4 μL
Forward Primer	1 μL
Reverse Primer	1 μL
rTaq	0.25 μL
DNA	1 μL

## Data Availability

All data have been included in the manuscript.
